# Lowered circulating aspartate is a metabolic feature of human breast cancer

**DOI:** 10.18632/oncotarget.5409

**Published:** 2015-10-01

**Authors:** Guoxiang Xie, Bingsen Zhou, Aihua Zhao, Yunping Qiu, Xueqing Zhao, Lana Garmire, Yurii B. Shvetsov, Herbert Yu, Yun Yen, Wei Jia

**Affiliations:** ^1^ Center for Translational Medicine, and Shanghai Key Laboratory of Diabetes Mellitus, Department of Endocrinology and Metabolism, Shanghai Jiao Tong University Affiliated Sixth People's Hospital, Shanghai, China; ^2^ University of Hawaii Cancer Center, Honolulu, HI, USA; ^3^ Department of Medical Oncology and Therapeutic Research, City of Hope National Medical Center, Duarte, CA, USA; ^4^ Albert Einstein College of Medicine, Yeshiva University, Bronx, NY, USA; ^5^ Nutrition Research Institute, University of North Carolina at Chapel Hill, North Carolina Research Campus, Kannapolis, NC, USA; ^6^ Taipei Medical University, Taipei, Taiwan

**Keywords:** breast cancer, metabolomics, aspartate, diagnosis, multivariate analysis

## Abstract

Distinct metabolic transformation is essential for cancer cells to sustain a high rate of proliferation and resist cell death signals. Such a metabolic transformation results in unique cellular metabolic phenotypes that are often reflected by distinct metabolite signatures in tumor tissues as well as circulating blood. Using a metabolomics platform, we find that breast cancer is associated with significantly (*p* = 6.27E-13) lowered plasma aspartate levels in a training group comprising 35 breast cancer patients and 35 controls. The result was validated with 103 plasma samples and 183 serum samples of two groups of primary breast cancer patients. Such a lowered aspartate level is specific to breast cancer as it has shown 0% sensitivity in serum from gastric (*n* = 114) and colorectal (*n* = 101) cancer patients. There was a significantly higher level of aspartate in breast cancer tissues (*n* = 20) than in adjacent non-tumor tissues, and in MCF-7 breast cancer cell line than in MCF-10A cell lines, suggesting that the depleted level of aspartate in blood of breast cancer patients is due to increased tumor aspartate utilization. Together, these findings suggest that lowed circulating aspartate is a key metabolic feature of human breast cancer.

## INTRODUCTION

Breast cancer remains to be one of the most commonly diagnosed and death-related cancers in women in the United States, resulting in an estimated 40,730 new deaths in 2015 [1–3]. The long-term survival of women with breast cancer depends on the stage of disease at the time of diagnosis: the 5-year survival rate is 99% for localized disease, 85% for regional stage, and 25% for distant-stage tumor [2]. Attempts to reduce breast cancer deaths have therefore relied greatly on early cancer detection and treatment. The most widely used screening method for breast cancer is mammography, with the sensitivity of the method ranging from 54% to 77% [4]. Despite the fact that image resolution continues to improve through the use of digital technology, tumors less than 5 mm are difficult to detect [5]. Other imaging methods such as thermography and magnetic resonance imaging are frequently used, but equally insensitive [6]. As the need for a screening test that would ideally be noninvasive, highly sensitive and specific continues to increase, considerable efforts have been devoted to search for biomarkers for early diagnosis of breast cancer.

Metabolomics has recently become a new driving force in cancer biology research shown some promise in identifying key metabolic pathways in various types of cancers [7–13]. Recent metabolomic studies of breast cancer have provided important metabolic signatures in serum, plasma [14–17], and tissue [18] that differentiate breast cancer from healthy controls. However, the significantly differential pathways and metabolites identified are not consistent among these studies, primarily due to the inter-individual variability of patients and the different analytical and clinical protocols used in various studies [16, 19, 20]. Moreover, none of the previous studies have evaluated selectivity of panels for breast cancer versus other malignancies.

Here, we report a metabolomics study aimed to identify distinct metabolite signatures of breast cancer patients. Plasma and sera samples from breast cancer patients and healthy controls were profiled using liquid chromatography time of flight mass spectrometry (LC-TOFMS) and gas chromatography-time of flight mass spectrometry (GC-TOFMS) coupled with bioinformatics tools. The initial training study was conducted with the use of 35 primary breast cancer plasma samples and 35 control samples, and validated with 103 primary breast cancer plasma samples and 183 primary breast cancer serum samples. Breast cancer tissue specimens and cell lines were also used to support the unique metabolic feature identified in the study.

## RESULTS

### Plasma metabolite profiling of breast cancer patients

**Figure 1:**
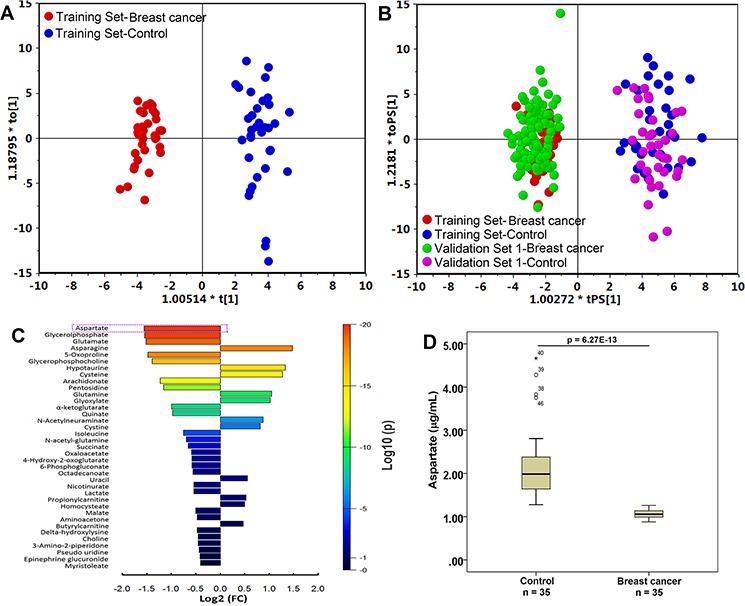
Metabolite profiles of breast cancer patients and healthy controls are significantly different **A.** The scores plot of the OPLS-DA model of the training group. The OPLS-DA model was constructed using the plasma data from 35 patients (red dots) and 35 healthy controls (blue dots). **B.** The OPLS-DA prediction model of breast cancer. An OPLS-DA model was constructed using the plasma data from 35 breast cancer patients (red dots) and 35 healthy controls (blue dots) (the “training set”); this model was then used to predict breast cancer of a group of 144 samples including 103 breast cancer patients (green dots) and 41 healthy controls (purple dots) that were not used in the construction of the model (Validation Set 1, the “testing set”). **C.** Bar plot of metabolite differences between breast cancer patients (*n* = 35 in the Training Set) and healthy controls (*n* = 35 in the Training Set). A fold change (FC) value was calculated for each metabolite by taking the ratio of the mean intensities in breast cancer patients and the healthy controls. Each bar representing an FC value was colored to indicate its corresponding *p*-value and thereby specify the statistical significance in all subjects (see color scale). **D.** Box plot of aspartate in distinguishing breast cancer (*n* = 35) from healthy control (*n* = 35).

Supplementary Figure S1 schematically shows the design and the data flow for discovery and validation of important metabolic signatures of breast cancer. A total of 225 metabolites were annotated from the detected spectral features of samples from Training Set (Table 1 and Supplementary Table S1), of which 102 metabolites (45.3%, 69 metabolites from GC-MS and 33, from LC-MS) were validated with reference standards while the others were annotated by comparing with the available databases including the NIST library and Human Metabolome Database (HMDB) (Supplementary Table S2). PCA was performed to assess the separation tendency between groups based on the 225 annotated metabolites in the Training Set samples including 35 breast cancer patients and 35 age-matched (but not ethnicity-matched) healthy controls from City of Hope National Medical Center (City of Hope). As shown in Supplementary Figure S2, there was a separation between healthy controls and breast cancer. Further, an OPLS-DA model with one predictive component and two orthogonal component (R2X(cum) = 0.140, R2Y(cum) = 0.861, Q2Y(cum) = 0.717) was constructed with satisfactory discriminating ability (Figure 1A). To account for metabolite variation due to age and racial differences between study participants, age and race were included as independent variables in the OPLS-DA model. A permutation test was performed 200 times on the OPLS-DA model including correlation coefficient between the original Y and the permuted Y versus the cumulative R2 and Q2, with the regression line shown in Supplementary Figure S3. The intercept (R2 and Q2 when the correlation coefficient is zero), which represents the extent of overfitting, is rather small (R2 = 0.51 and Q2 = 0.19) and the model is satisfactory. The performance of this OPLS-DA model was further tested in another group of 103 breast cancer patients and 41 healthy controls (Validation Set 1) and all test samples were correctly classified by the OPLS-DA model established with the 70 training samples. Moreover, the OPLS-DA model of 225 metabolites showed distinct metabolite profiles of breast cancer patients at Stages I - II and the patients at stages III - IV, from the healthy controls, with satisfactory modeling and predictive abilities using one predictive component and three orthogonal components (Supplementary Figure S4).

**Table 1:** Demographic and clinical pathological characteristics of study population

		Training Set		Validation Set 1		Validation Set 2		Validation Set 3		Sample Set 1	Test Set 1	Test Set 2
		Breast cancer	Healthy control	Breast cancer	Healthy control	Breast cancer	Healthy control	Breast cancer	Healthy control	Breast cancer	Gastric cancer	Colorectal cancer
**Biospecimen**		Plasma	Plasma	Plasma	Plasma	Serum	Serum	Serum	Serum	Tissue	Serum	Serum
**Number of participants**		35	35	103	41	103	31	80	70	20	114	101
**Age (median, range)**		40, 31–45	38, 35–40	58, 46–73	30, 21–35	52, 32–72	36, 18–49	48, 36–78	58, 35–76	65, 46–75	58, 27–80	60, 24–83
**TNM stage-I**		1		18				10		1	27	24
**TNM stage-II**		16		34		52		45		10	18	45
**TNM stage-III**		16		33		51		25		8	59	27
**TNM stage-IV**		2		18						1	10	5
**ER status (positive/negative/unknown)**		12/20/3		65/34/4								
**PR status (positive/negative/unknown)**		9/23/3		55/44/4								
**HER-2 status (positive/negative/unknown)**		11/23/1		39/57/7								
**Race**	Asian	4		14		14		80	70		114	101
	Black	3	8	6	5	6	5					
	White	23	17	77	20	69	21			20		
	Latino		10		16		5					
	Native			1		1						
	Others	5		5		13						

Using the VIP values (VIP > 1) derived from the OPLS-DA model and the *p* values (*p* < 0.05) from the Mann-Whitney test, a total of 31 metabolites were selected as differential variables between breast cancer patients and controls from the Training Set with detailed statistics of the area under the ROC curves (AUC), and the corresponding sensitivity and specificity (Table 2). Participants’ age and race, with VIP values of 0.98 and 0.33, respectively, did not pass the VIP threshold.

**Table 2:** Summary of differentially expressed plasma metabolites in patients of breast cancer relative to healthy controls (see Table 1 for detailed patient numbers) with detailed statistics of the area under the ROC curves (AUC), and the corresponding sensitivity and specificity for each of the 31 plasma metabolites

Pathway	Compound	Training Set						Validation Set 1				
		VIP^a^	FC^b^	*P*^c^	AUC	Sensitivity (%)	Specificity (%)	FC^b^	*P*^c^	AUC	Sensitivity (%)	Specificity (%)
**Amino Acid Metabolism**	Aspartate	3.13	0.34	6.27E-13	1.000 (1.000–1.000)	100.0	100.0	0.47	3.99E-16	0.935 (0.884–0.987)	85.4	95.1
	Glutamate	2.93	0.35	2.25E-12	0.988 90.971–1.000)	97.1	91.4	0.47	1.32E-15	0.928 (0.872–0.983)	75.6	98.1
	5-Oxoproline	3.33	0.36	6.62E-12	0.977 (0.951–1.000)	97.1	88.6	0.49	4.50E-14	0.904 (0.839–0.968)	78.0	96.1
	Isoleucine	1.9	0.59	1.92E-04	0.759 (0.647–0.842)	57.1	85.7	0.71	5.78E-04	0.684 (0.586–0.782)	73.2	62.1
	N-acetyl-glutamine	1.48	0.62	5.91E-04	0.739 (0.615–0.862)	74.3	74.3	0.78	1.20E-02	0.634 (0.537–0.731)	80.5	48.5
	Aminoacetone	1.26	0.72	1.91E-02	0.663 (0.530–0.795)	68.6	68.6	0.84	8.96E-02	0.591 (0.477–0.705)	51.2	80.6
	Delta-hydroxylysine	1.18	0.72	1.97E-02	0.662 (0.533–0.791)	80.0	51.4	0.81	4.38E-02	0.608 (0.503–0.713)	43.9	77.7
	Cystine	2.17	1.76	5.47E-05	0.780 (0.671–0.889)	77.1	74.3	2.94	2.05E-14	0.909 (0.854–0.964)	87.4	82.9
	Glutamine	2.57	2.07	3.77E-07	0.853 (0.765–0.942)	74.3	88.6	2.48	1.28E-11	0.862 (0.782–0.942)	96.1	73.2
	Cysteine	3.14	2.42	1.40E-09	0.921 (0.858–0.984)	94.3	77.1	2.57	3.48E-12	0.872 (0.802–0.942)	85.4	78.0
	Hypotaurine	3.41	2.51	3.17E-10	0.937 (0.880–0.994)	80	100	2.71	4.48E-13	0.887 (0.823–0.951)	75.7	87.8
	Asparagine	3.66	2.78	6.10E-12	0.978 (0.951–1.000)	97.1	91.4	2.92	2.79E-14	0.907 (0.847–0.967)	81.6	87.8
**Lipid Metabolism**	Glycerol phosphate	3.12	0.34	9.63E-13	0.996 (0.988–1.000)	100	94.3	0.44	1.39E-18	0.971 (0.935–1.000)	95.1	93.2
	Glycero phospho choline	3.06	0.38	5.81E-11	0.955 (0.909–1.000)	97.1	88.6	0.49	1.79E-14	0.910 (0.860–0.960)	95.1	87.4
	Arachidonate	2.33	0.43	4.43E-09	0.908 (0.841–0.974)	88.6	80.0	0.51	7.79E-13	0.883 (0.824–0.923)	85.4	79.6
	Octadecanoate	1.09	0.68	5.28E-03	0.694 (0.569–0.819)	80.0	54.3	0.7	2.76E-04	0.695 (0.602–0.787)	53.7	76.7
	Nicotinurate	1.88	0.69	7.02E-03	0.687 (0.559–0.816)	45.7	97.1	0.74	3.31E-03	0.657 (0.554–0.761)	65.9	66.0
	Choline	1.15	0.73	2.30E-02	0.658 (0.528–0.788)	91.4	40.0	0.81	4.19E-02	0.609 (0.504–0.714)	46.3	76.7
	Myristoleate	1.11	0.76	4.40E-02	0.640 (0.510–0.770)	65.7	62.9	0.75	3.36E-03	0.657 (0.558–0.756)	58.5	71.8
	Butyryl carnitine	1.25	1.38	1.94E-02	0.662 (0.532–0.793)	74.3	65.7	1.71	9.20E-06	0.737 (0.654–0.820)	62.1	90.2
	Propionyl carnitine	1.61	1.44	8.66E-03	0.682 (0.557–0.808)	40.0	94.3	1.58	1.21E-04	0.706 (0.614–0.797)	55.3	80.5
**Carbohydrate Metabolism**	α-ketoglutarate	1.79	0.50	1.34E-06	0.836 (0.723–0.949)	77.1	94.3	0.8	2.41E-02	0.621 (0.486–0.756)	53.7	93.2
	Oxaloacetate	1.58	0.67	3.38E-03	0.704 (0.577–0.830)	62.9	77.1	0.81	4.47E-02	0.607 (0.503–0.711)	68.3	54.4
	4-Hydroxy-2-oxoglutarate	1.26	0.67	3.93E-03	0.700 (0.573–0.828)	71.4	68.6	0.8	2.64E-02	0.619 (0.513–0.725)	61.0	68.9
	Lactate	1.92	0.69	7.02E-03	0.687 (0.559–0.816)	42.9	100.0	0.66	2.43E-05	0.726 (0.626–0.825)	61.0	82.5
	Malate	1.59	0.71	1.18E-02	0.675 (0.546–0.804)	48.6	91.4	0.71	6.91E-04	0.682 (0.582–0.781)	58.5	73.8
	Glyoxylate	1.93	2.03	7.38E-07	0.844 (0.748–0.940)	91.4	74.3	2.07	1.11E-08	0.806 (0.722–0.889)	76.7	75.6
**Nucleotide Metabolism**	Pentosidine	1.89	0.45	2.58E-08	0.887 (0.812–0.961)	97.1	65.7	0.48	4.99E-15	0.918 (0.871–0.964)	80.5	91.3
	Uracil	1.29	1.47	5.28E-03	0.694 (0.568–0.820)	54.3	85.7	1.42	2.55E-03	0.661 (0.565–0.758)	57.3	75.6
**Others**	Quinate	2.22	0.51	1.91E-06	0.831 (0.732–0.930)	77.1	85.7	0.92	4.14E-01	0.544 (0.420–0.667)	48.8	79.6
	Epinephrine glucuronide	1.01	0.75	3.90E-02	0.643 (0.610–0.776)	71.4	65.7	0.88	2.08E-01	0.567 (0.449–0.686)	39.0	90.3

a Variable importance in the projection (VIP) was obtained from OPLS-DA model with a threshold of 1.0.

b The Fold change (FC) with *a* value larger than 1.0 indicates a significantly higher level of the plasma metabolite in patients while a FC value lower than 1.0 indicates a lower level, relative to healthy controls.

c *p* means *p* value obtained from nonparametric Mann-Whitney test.

Using the Kyoto Encyclopedia of Genes and Genomes (KEGG) database, key metabolic pathways dysregulated in breast cancer patients were identified as the TCA cycle, amino acid metabolism, lipid metabolism and nucleotide metabolism (Table 2). Among these, the amino acid metabolic pathway had the most differential metabolites and showed a profound change.

In addition case vs. control comparisons, breast cancer cases were compared between early (I-II) and late (III-IV) stage of the disease, with a list of differential metabolites given in Supplementary Table S3.

### Circulating metabolite signatures of breast cancer

Among the above 31 candidate metabolites, aspartate is the most significant differential metabolite that markedly decreased in breast cancer samples with a fold change of 0.34 (*p* = 6.27E-13) in the Training Set (Figure 1C and 1D). The ROC curve analysis revealed that aspartate has the highest value of the area under the curve (AUC) of 1.000, and specificity and sensitivity (Table 2), which showed excellent discriminating power in distinguishing breast cancer cases from healthy controls. A logistic regression analysis of the 31 metabolites conducted using the Training Set also showed that aspartate had the strongest association with a decrease in the risk of breast cancer in Training set (*p* = 5.3E-05). The box plot established with plasma aspartate levels (Supplementary Figure S5) showed that breast cancer patients at all four stages had a significantly lower aspartate abundance than the healthy controls.

### Validation of aspartate with different patient cohorts

The 31 candidate metabolites were further tested for differences and cancer risk association using Validation sets 1, 2 and 3. Five of these 31 metabolites, aspartate, glycerolphosphate, 5-oxoproline, arachidonate, and isoleucine were all significantly decreased in breast cancer patients in Validation Set 1, 2 and 3, among which aspartate was the most significant (Table 3). A logistic regression analysis of the 31 metabolites was conducted using the three Validation Sets and showed that aspartate had the strongest association with a decrease in the risk of breast cancer in Validation set 1 (*p* = 1.7E-02, odds ratio: 0.77; 95% confidence interval (CI): 0.625 to 0.955), Validation Set 2 (*p* = 2.0E-03, odds ratio: 0.163; 95% CI: 0.053 to 0.501), and Validation Set 3 (*p* = 2.5E-05, odds ratio: 0.895; 95% CI: 0.850 to 0.943).

**Table 3:** Summary of differentially expressed blood metabolites in patients of breast cancer relative to healthy controls from the Training Set and Validation Set 1, 2 and 3 (see Table 1 for detailed patient numbers)

Compound	Training Set			Validation Set 1			Validation Set 2			Validation Set 3		
	FC^a^	p^b^	AUC	FC^a^	p^b^	AUC	FC^a^	p^b^	AUC	FC^a^	p^b^	AUC
Aspartate	0.34	6.27E-13	1.000 (1.000–1.000)	0.47	3.99E-16	0.935 (0.884–0.987)	0.29	2.10E-32	0.984 (0.962–1.000)	0.43	4.81E-19	0.986 (0.962–1.000)
Glycerol phosphate	0.34	9.63E-13	0.996 (0.988–1.000)	0.44	1.39E-18	0.971 (0.935–1.000)	0.50	1.00E-17	0.895 (0.818–0.972)	0.46	2.02E-11	0.969 (0.939–0.998)
5-Oxoproline	0.36	6.62E-12	0.977 (0.951–1.000)	0.49	4.50E-14	0.904 (0.839–0.968)	0.52	4.22E-14	0.967 (0.938–0.996)	0.45	7.69E-17	0.945 (0.907–0.982)
Arachidonate	0.43	4.43E-09	0.908 (0.841–0.974)	0.51	7.79E-13	0.883 (0.824–0.923)	0.60	2.46E-12	0.968 (0.939–0.996)	0.27	5.46E-13	0.918 (0.868–0.968)
Isoleucine	0.59	1.92E-04	0.759 (0.647–0.842)	0.71	5.78E-04	0.684 (0.586–0.782)	0.80	2.30E-05	0.864 (0.789–0.938)	0.58	9.79E-07	0.870 (0.805–0.934)

a The Fold change (FC) with *a* value larger than 1.0 indicates a significantly higher level of the plasma/serum metabolite in patients while a FC value lower than 1.0 indicates a lower level, relative to healthy controls.

b *p* means *p* value obtained from nonparametric Mann-Whitney test.

**Figure 2:**
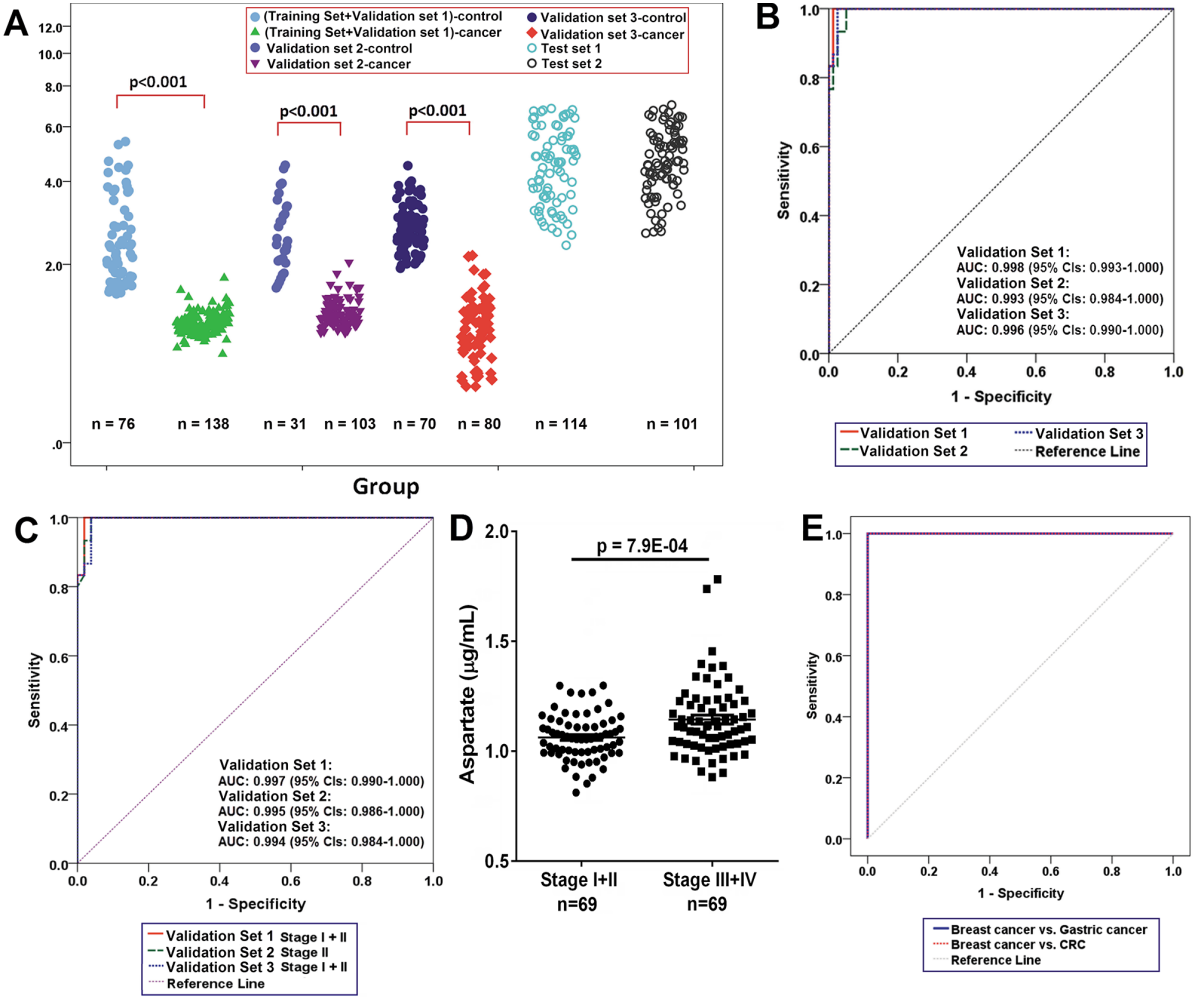
Lowered circulating aspartate is a metabolic feature of human breast cancer **A.** Distributions of aspartate concentration in different samples. *P*-value over a group denotes statistical significance of differences between each group member and healthy controls. **B.** The ROC curves in the breast cancer samples from the Validation Set 1, Validation set 2, and Validation Set 3 using aspartate. **C.** The ROC curves in the breast cancer samples of Stage I+II from the Validation Set 1, Validation set 2, and Validation Set 3 using aspartate. **D.** Plasma aspartate levels in patients at Stages I and II (*n* = 69) are significantly different from the patients at Stages III and IV (*n* = 69) from the Training Set and validation Set 1. **E.** ROC curves in the breast cancer samples (*n*= 138) from the Training Set and validation Set 1 and in the gastric (*n* = 114) and colorectal (*n* = 101) cancer samples using aspartate.

In order to explore the potential of aspartate as a key differential metabolite, blood samples from the Training Set and Validation Sets 1, 2 and 3 were subjected to quantitative metabolic analysis. Bar plots constructed using the aspartate levels from plasma (Validation Set 1, Figure 2A) and sera (Validation Set 2 and 3, Supplementary Figure S6 and S7), show a marked decrease in cancer patients. ROC analysis reveals that aspartate has the highest accuracy (AUC = 1.000) with sensitivity of 100% and specificity of 100% (Table 4) at a cutoff value of 1.27 μg/mL using the quantitative data of aspartate in the Training set. The parameters estimated from the Training set were also used to predict the probability of being diagnosed breast cancer patients for the independent Validation Sets. In the ROC analysis (Figure 2B), the AUC of aspartate in plasma samples from Validation set 1 was 0.998 (95% CI: 0.993–1.000), with a sensitivity of 100% and a specificity of 98.7%. The AUC of serum aspartate from Validation set 2 was 0.993 (95% CI: 0.984–1.000), with a sensitivity of 100% and a specificity of 94.9%, while for the serum samples from Shanghai (validation set 3), the AUC was 0.996 (95% CI: 0.990–1.000), with a sensitivity of 100% and a specificity of 97.5%. Notably, the ability of the aspartate to differentiate between early stage breast cancer patients and healthy controls is also significant (Figure 2C), with an overall accuracy of 99% for early-stage tumors. Moreover, the levels of plasma aspartate are significantly different between patients at Stages I-II and at Stages III-IV (*p* = 7.90E-04, Figure 2D).

**Table 4:** Diagnostic accuracy of aspartate for the diagnosis of breast cancer

		Diagnostic model
Training set	AUC (95% confidence interval)	1.000 (1.000–1.000)
	Cut-off value (μg/ml)	1.27
	Sensitivity	100%
	specificity	100%
Validation set 1	AUC (95% confidence interval)	0.998 (0.993–1.000)
	Sensitivity	100%
	specificity	98.7%
Validation set 2	AUC (95% confidence interval)	0.993 (0.984–1.000)
	Sensitivity	100%
	Specificity	94.9%
Validation Set 3	AUC (95% confidence interval)	0.996 (0.990–1.000)
	Sensitivity	100%
	Specificity	97.5%

### Selectivity of aspartate for breast cancer versus other cancers

Aspartate was used to classify a set of cases with other two common cancers, colorectal (*n* = 101, Test Set 1) and gastric (*n* = 114, Test Set 2). Aspartate correctly differentiated all of colorectal cancers and gastric cancers from the breast cancer (Figure 2E).

### Aspartate in breast cancer tissue samples and breast cancer cell lines

**Figure 3:**
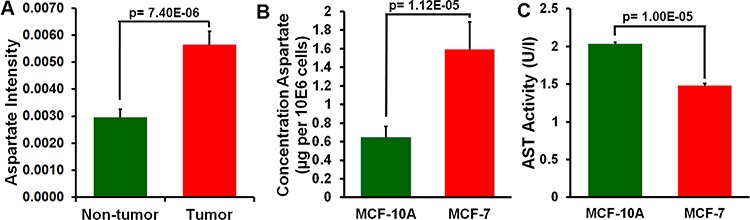
Analysis of aspartate in breast cancer tissue and adjacent non-tumor tissue and in MCF-7 and MCF-10A cells **A.** Aspartate level in breast cancer tissue and adjacent non-tumor tissue (mean ± SEM, *n* = 20). **B.** Aspartate level in MCF-7 and MCF-10A cells (mean ± SEM, *n* = 5). **C.** Aspartate aminotransferase (AST) activity in MCF-7 cells was lower than in MCF-10A cells (mean ± SEM, *n* = 3).

To determine whether the aspartate decrease in blood of breast cancer patients has any biological relevance, we profiled 20 pairs of breast tumor tissue and the adjacent non-tumor tissue (Sample Set 1) using GC-TOFMS and LC-TOFMS. Aspartate was found elevated by 1.92 fold in the tumor tissue with a *p* value of 7.4E-06 (Figure 3A), and the levels of aspartate-related metabolites, such as asparagine and several nucleosides and nucleobases including uridine, uracil, guanosine, orotidine, dihydrouracil, hypoxanthine, and 8-hydroxy-deoxyguanosine, were all elevated in breast tumor tissues (Supplementary Table S4).

We also measured aspartate levels in breast cancer cell lines of MCF-7 (*n* = 5) and the control cell line of MCF-10A (*n* = 5). Significantly increased aspartate concentration was found in the MCF-7 cells relative to the MCF-10A cells (*p* = 1.12E-05, Figure 3B). Additionally, the AST activity in the MCF-7 cells was lower than in the MCF-10A cells (*n* = 3, *p* = 1.00E- 05, Figure 3C).

## DISCUSSION

Breast cancer is the most common cancer in women. While genetic alterations have been extensively characterized in breast cancer, the changes in metabolism that occur downstream from genomic and proteomic alterations have not been characterized in detail to date. Since blood is in contact with virtually all tissues in the human body and is considered to reflect in a dynamic way the pathophysiological status, serum/plasma metabolomic changes are of particular importance with diagnostic value for early cancer detection [21].

We used combined LC-TOFMS and GC-TOFMS to profile serum, plasma and breast cancer tissue metabolites [12, 22], providing an unprecedented number of identified metabolites for explaining the biological variations associated with pathophysiological conditions [23] and obtaining cross-validated results as well. The current study revealed a lowered blood (plasma or serum) aspartate level in breast cancer patients, and an increased level in breast tumor tissues compared to adjacent non-tumor tissues and in breast cancer cells (MCF-7) as compared to MCF-10A cells. Since the characteristic metabolite expressions may be associated with age and race, we performed logistic regression analysis after adjustment of age and race and the aspartate was sensitive at detecting breast cancer that were adjusted for age and race. We also compared the levels of aspartate among breast cancer patients with different races, and found that aspartate is at the similar level among the groups.

Two of the most commonly diagnosed cancers, gastric and colorectal cancers, were used to evaluate the selectivity of aspartate for breast cancer. Aspartate was highly selective for breast cancer when compared with gastric and colorectal cancer, suggesting that aspartate can be breast cancer specific. Due to the fact that not all the gastric and colorectal cancer patients are gender- and age-matched, we carefully selected 35 age-matched female patients with gastric cancer and colorectal cancer, respectively, to test the specificity of aspartate again. As a result, aspartate was able to correctly differentiate all of the colorectal cancers and gastric cancers from the breast cancer (Supplementary Figure S8).

Aspartate is a non-essential amino acid, produced from oxaloacetate by a transamination process. It participates in urea cycle to facilitate the removal of ammonia, functions as a substrate of de novo biosynthesis of pyrimidine, plays a role in translocating nicotinamide adenine dinucleotide (NADH) (produced from glycolysis) into mitochondria across inner mitochondria membrane for oxidative phosphorylation by aspartate-malate shuttle, and can be converted to alanine and then undergoes gluconeogenesis. The de novo pyrimidine biosynthesis activity was found elevated by 4.4 folds in the MCF-7 cells compared to that in the MCF-10A cells [24], which may account for the higher aspartate levels in the MCF-7 cells. The high rate of tumor growth requires increased biosynthesis of nucleic acids [25], and thus, increased utilization of aspartate by tumor cells, leading to the decreased level of aspartate and oxaloacetate in blood. It was consistent with the results obtained by Proenza *et al*. that the plasma, blood and blood cell aspartate were all decreased in 16 female breast cancer patients [19]. However, our results were inconsistent with several other reports. It was reported that the serum aspartate level was increased in breast cancer patients (*n* = 41) without significance compared to healthy controls (*n* = 9) (FC = 1.50) [16]. The plasma total amino acids, essential amino acids, branched chain amino acids, aromatic amino acids and gluconeogenic amino acids were also increased in breast cancer patients (*n* = 22) compared to healthy controls (*n* = 6) [20]. However, all these results were obtained with small number of participants without validation.

The high aspartate level in breast cancer cell line and low level in human breast epithelial cell line demonstrate its close association with the down-regulated AST activity (Figure 3C), an enzyme involved in the transfer of an amino group from aspartate to α-ketoglutarate to produce oxaloacetate and glutamate. AST, essential for the transformation and growth of mammary epithelial cells, was proposed as a valid target for the anti-breast cancer agents [26], supporting the biological relevance of aspartate as a metabolic feature of breast cancer. In addition to AST, aspartate level is regulated by asparagine synthetase, an enzyme that generates asparagine from aspartate [27]. It has been found that asparagine synthetase was overexpressed under glucose-deprived condition in the pancreatic cancer cells with a protective capability against apoptosis for the cancer cells [27]. It has been observed that overexpression of insulin-like growth factor (IGF) 1 and 2 (an essential regulator of breast cancer development) in the MCF-7 breast cancer cells also leads to an increased expression of asparagine synthetase [28]. The elevated asparagine and decreased aspartate observed in this breast cancer metabolomics study may be a result of an overexpression of asparagine synthetase.

The metabolite signature obtained in this study cannot readily stratify breast cancer patients at each stage, although it can differentiate breast cancer patients at Stages I and II from the patients at Stages III and IV. The heterogenic background of breast cancer such as estrogen receptor (ER), progesterone receptor (PR), and human epidermal growth factor receptor (HER2), didn't seem to complicate the metabolite profile of breast cancer. It is likely that different molecular subtypes may share a common metabolite signature, resulting in a unique metabolic feature of human breast cancer regardless of its heterogeneity. The fact that the aspartate offered high accuracy in a heterogeneous validation set that contained different molecular subtypes underscores the potential utility of this metabolic feature of breast cancer.

In summary, we used mass spectrometry-based metabolomics approach to characterize the serum and plasma metabolite signature of breast cancer patients. The results reveal a potentially important metabolic feature of breast cancer characterized by a depleted circulating aspartate and elevated aspartate levels in tumor tissue and cells.

## MATERIALS AND METHODS

### Patient populations

The population used in this study comprised 321 breast cancer patients, 114 gastric cancer patients, 101 colorectal cancer patients and 177 healthy controls (Table 1) and were obtained from multiple sources (Supplementary Table S1). The breast cancer patients were newly diagnosed and were not recurrent or on any medication prior to sample collection. Patient characteristics, staging of disease and other parameters are shown in Table 1. Control samples were collected from a total of 177 healthy volunteers using the same sample collection protocol. Supplementary Figure S1 schematically shows the design and the data flow for identifying important metabolic signatures of breast cancer.

This study was approved by the institutional review boards of Shanghai Jiao Tong University Affiliated Ruijin Hospital and City of Hope National Medical Center, and all participants signed an informed consent before they participated in the study.

### Collection and storage of blood serum and plasma

Fasting blood samples were collected in the morning before breakfast from all the participants. Plasma and serum specimens were obtained and placed into clean tubes and immediately stored within two hours of collection at −80°C until analysis.

### Tissue samples

Twenty pairs of frozen breast cancer tissues of Stage I (*n* = 1), Stage II (*n* = 10), Stage III (*n* = 8), and Stage IV (*n* = 1) were purchased from Biochain Institute in CA, USA. Each pair of tissues consisted of breast tumor tissue and the adjacent non-tumor tissue from the same patient. Tissues were collected under an IRB approved protocol (Biochain Institute) and tissues used in study did not need additional ethical approval.

### Metabolite profiling

LC-TOFMS and GC-TOFMS were used for the metabolomics profiling of all samples in the study. The profiling procedure (sample preparation, metabolite separation and detection, metabolomic data preprocessing, metabolite annotation, and statistical analysis) was performed following our previously published protocols with minor modifications [12, 22, 29–33]. Details of plasma, serum and tissue sample preparation and LC/GC-MS analysis are provided in Supplementary Methods.

Results obtained in our study revealed that aspartate is the most significant key differential metabolite; we then quantified aspartate in all samples using an ultra-performance liquid chromatography (UPLC) coupled with an ACQUITY TQ tandem mass spectrometry (Waters, Milford, MA, USA).

### Data analysis and statistics

All annotated metabolites from GC-TOFMS and LC-TOFMS datasets were combined and exported to SIMCA-P+ 12.0 software (Umetrics, Umeå, Sweden) for multivariate statistical analysis [22, 34]. Principal component analysis (PCA, an unsupervised multivariate statistical analysis) and orthogonal partial least squares-discriminant analysis (OPLS-DA, a supervised multivariate analysis for identification of key variances between different datasets) [35] were performed to discriminate between the breast cancer patients and healthy controls. Using the training dataset and the threshold of variable importance in the projection (VIP, value >1) from the 7-fold cross-validated OPLS-DA model and the *p*-value (<0.05) from Mann-Whitney test, a panel of metabolites responsible for the difference in the metabolic profiles of cancer cases and controls were obtained. These differential metabolites were then evaluated in Validation sets 1–3 to test metabolite differences between cancer cases and controls, and logistic regression adjusted for participants’ race and age at blood draw to examine the association between metabolites and breast cancer risk. The corresponding fold change was calculated to show how these selected differential metabolites varied in the cancer samples relative to the healthy controls. In addition, receiver operating characteristic (ROC) curve analysis was conducted using the SPSS software (IBM SPSS Statistics 19, USA) [11, 36]. This analysis was performed to identify the optimal threshold values for key differential metabolites. Bar plots of the metabolites were constructed using the R software package (http://www.r-project.org). We regarded *p* values of < 0.05 as significant.

### Aspartate levels in MCF-10A and MCF-7 cells

MCF-7 breast adenocarcinoma cells and MCF-10A non-tumorigenic epithelial cells used as the control cell line were obtained from the American Type Culture Collection (ATCC) in VA, USA and had been authenticated by STR profiling. The aspartate levels in the MCF-7 and MCF-10A were quantitatively measured by UPLC coupled with an ACQUITY TQ tandem mass spectrometry (Waters, Milford, MA). Briefly, cell samples were homogenized in a bullet blender (Next Advance) in 500 μl mixture of chloroform, methanol and water (1:2.5:1, v/v/v) containing ^13^C-labeled aspartate used as internal standard. The samples were then centrifuged at 13,000 rpm for 10 min at 4°C, and a 150-μl aliquot of the supernatant was transferred to an LC sampling vial. The deposit was re-homogenized with 500 μL of methanol, and after centrifuging, a 150-μL aliquot of supernatant was added to the same vial for drying prior to reconstitution with acetonitrile/H2O (6:4, v/v) to a final volume of 100 μl. A volume of 10 μL sample extraction was injected onto an Atlantis HILIC silica column (50 × 4.6 mm, 5 μm). The samples were analyzed with mobile phase A (10% acetonitrile in water) and mobile phase B (10% water in acetonitrile), both containing 10 mM ammonium formate and 0.125% formic acid. The flow rate was 1 mL/min and the column temperature was 40°C. The gradients were 0 min, 95% B; 0.05 min, 95% B; 0.4 min, 90% B; 1.5 min, 90% B; 1.55 min, 35% B; 3.5 min, 35% B; 3.55 min, 95% B; and 6 min, 95% B. The mass spectrometry was operated in the positive electrospray mode using a capillary voltage of 3 kV and an extractor voltage of 3 V. The source and desolvation temperatures were maintained at 120°C and 250°C, respectively. The gas flow rates of cone, desolvation and collision were set to 60 L/h, 500 L/h and 0.1 mL/min, respectively. The multiple reaction monitoring (MRM) transitions for aspartate and ^13^C-labeled aspartate were optimized and shown in the Supplementary Table S5. UPLC-MS raw data obtained with positive mode were analyzed using QuanLynx applications manager version 4.1 (Waters, Manchester, UK). The concentrations of metabolites were expressed in μg per 10^6^ cells. A Student's *t*-test was used to investigate differences between the groups in aspartate measurement.

### Aspartate aminotransferase activity assay

The enzyme activity of aspartate aminotransferase (AST) in the cell samples were measured and analyzed using AST activity assay kit (BioVision, Milpitas, CA) according to the manufacturer's instructions.

## SUPPLEMENTARY METHODS


