# Supplementary material for: Lowered circulating aspartate is a metabolic feature of human breast cancer
Source: Oncotarget. 2015 Oct 1;6(32):33369–81. doi: 10.18632/oncotarget.5409 (PMC4741772; doi:10.18632/oncotarget.5409)
Supplement: Supplementary file 1 [file oncotarget-06-33369-s001.pdf]

SUPPLEMENTARY METHODS

Plasma and serum sample preparation and analysis by gc-tofms

Plasma/serum metabolite extraction and analysis were performed following our previously published procedure [1, 2] with minor modifications. A 50 µL aliquot of plasma/serum sample was spiked with two internal standard solutions (10 µl *p*-chlorophenylalanine in water, 0.1 mg/mL; 10 µL heptadecanoic acid in methanol, 1 mg/mL). The mixed solution was extracted with 175 µL of methanol: chloroform (3:1) and vortexed for 30 seconds. After storing for 10 minutes at –20°C, the samples were centrifuged at 13,000 rpm for 10 minutes. An aliquot of 200 µL supernatant was transferred to a glass sampling vial to vacuum dry at room temperature. The residue was derivatized using a two-step procedure. First, 50 µL methoxyamine (15 mg/mL in pyridine) was added to the vial and kept at 30°C for 90 minutes. After adding 10 µL C10-C40 (all even alkanes, 12.5 µg/mL) as retention index, 50 µL N,O-bis-(trimetylsilyl) trifluoroacetamide (BSTFA) (1% trimethylchlorosilane, TMCS) was added to the samples, before being derivatized at 70°C for 60 minutes.

Each 1 µL aliquot of the derivatized solution was injected in splitless mode into an Agilent 6890N gas chromatography coupled with a Pegasus HT time-of-flight mass spectrometry (Leco Co., St. Joseph, MI, USA). To minimize systematic analytical deviations, each control sample was separated by 1 or 2 breast cancer samples. Breast cancer samples from different stages were also run evenly in the whole experiment. Separation was achieved on an Rxi-5 ms capillary column (Crossbond ® 5% diphenyl/95% dimethyl polysiloxane, Restek, PA, USA), with helium as the carrier gas at a constant flow rate of 1.0 mL/min. The temperatures of injection, transfer interface, and ion source were set to 260, 260, and 210°C, respectively. The GC temperature programming was set to 2 min isothermal heating at 80°C, followed by 10°C/min oven temperature ramped to 220°C, 5°C/min to 240°C, and 25°C/min to 290°C, and a final eight minute maintenance at 290°C. Electron impact ionization (70 eV) at full scan mode (*m/z* 40–600) was used, with an acquisition rate of 20 spectra/second in the TOFMS setting.

The data generated in the GC-TOFMS instrument were analyzed by the ChromaTOF software (v4.33, Leco Co, CA, USA). Using the statistic component, the aligned comma separated value (CSV) file can be obtained with sample information, peak information and peak intensity. Peak areas of unique mass were normalized to the internal standard. Compound identification was performed by comparing the mass fragments with NIST 05 Standard mass spectral databases in NIST MS search 2.0 (NIST, Gaithersburg, MD, USA) software with a similarity of more than 70% and reference standards (with retention time, or retention index if available in the library, as another parameter). Internal standards and any known artificial peaks, such as peaks caused by noise, column bleed and BSTFA derivatization procedure, were removed from the dataset before statistical analysis.

Tissue sample preparation and analysis by gc-tofms

Tissue sample preparation and analysis by GC-TOFMS was performed according to our published report [3, 4]. Approximately 50 mg of tissue samples were prepared using a two-step extraction. The tissue sample was first added with 50 µL of the first-step extraction solvent (chloroform : methanol : water = 1:2.5:1) and homogenized for 6 minutes in a bullet blender (Next Advance, Inc., BIOBOXTM). Then, the sample was extracted with another 250 µL of first-step extraction solvent, and centrifuged at 13,200 rpm for 20 minutes at 4°C. An aliquot of 100 µL supernatant was transferred to a GC sampling vial. At the second step, the deposit of tissue was extracted with 300 µL methanol. After centrifugation at 13,200 rpm for 20 minutes at 4°C, an aliquot of 100 µL supernatant was transferred to the same GC vial. After vortexing, the samples spiked with two internal standards (10 µL heptadecanoic acid of 1 mg/mL and 4-chlorophenylalanine of 0.3 mg/mL) were vacuum dried at room temperature. The residue was derivatized using a two-step procedure. First, 80 µL methoxyamine (15 mg/mL in pyridine) was added to the vial and kept at 30°C for 90 minutes. Then, 80 µL BSTFA (1%TMCS) was added to the samples, before being derivatized at 70°C for 60 minutes.

The rest of GC-TOFMS operations and statistical procedures were carried out as the GC-TOFMS analysis of plasma/serum samples.

Plasma and serum sample preparation and analysis by lc-tofms

Plasma/serum sample preparation and analysis with LC-TOFMS was performed according to our published report [2, 5, 6]. The plasma/serum samples were thawed and centrifuged at 13,000 rpm for 5 min. A volume of 100 µL supernatant was mixed with 400 µL mixture of methanol and acetonitrile (5:3) containing *p*-chlorophenylalanine as internal standard (5 µg/mL). The mixture was vortexed for 2 min, allowed to stand for 10 min, and then centrifuged at 13,000 rpm for 20 min; and the supernatant was used for LC-TOFMS analysis.

An Agilent HPLC 1200 system equipped with a binary solvent delivery manager and a sample manager (Agilent Corporation, Santa Clara, CA, USA) was used with chromatographic separations performed on a 4.6 × 150 mm 5 µm Agilent ZORBAX Eclipse XDB-C18 chromatography column. The LC elution conditions were optimized as follows: isocratic at 1% B (0–0.5 min), linear gradient from 1–20% B (0.5–9.0 min), 20–75% B (9.0–15.0 min), 75–100% B (15.0–18.0 min), isocratic at 100% B (18.0–19.5 min); linear gradient from 100% to 1% B (19.5–20.0 min) and isocratic at 1% B (20.0–25.0 min). Here, A = water with 0.1% formic acid and B = acetonitrile with 0.1% formic acid. The column was maintained at 30°C. A 10 µL aliquot sample was injected onto the column. Mass spectrometry was performed using an Agilent model 6220 MSD TOF MS equipped with a dual sprayer electrospray ionization source (Agilent Corporation, Santa Clara, CA, USA). The system was tuned for optimum sensitivity and resolution using an Agilent ESI-L low concentration tuning mix in both positive (ES+) and negative (ES-) electrospray ionization modes. Agilent API-TOF reference mass solution kit was used to obtain accurate mass time-of-flight data in both positive and negative mode operation. The TOF MS was operated under the following optimized conditions: (1) ES+ mode, capillary voltage 3500 V, nebulizer 45 psig, drying gas temperature 325°C, drying gas flow 11 L/min, and (2) ES- mode, similar conditions as ES+ mode except the capillary voltage that was adjusted to 3000 V. The TOF MS is calibrated routinely in ES+ and ES- modes using the Agilent ESI-L low concentration tuning mix. During metabolite profiling experiments, both plot and centroid data were acquired for each sample from 50 to 1,000 Da over a 25-min analysis time.

The resulting .d files were then centroided, deisotoped, and converted to mzData xml files using the MassHunter Qualitative Analysis Program (vB.03.01) (Agilent). Following the conversion, the xml files were analyzed using the open source XCMS package (v1.24.1) (http://metlin.scripps.edu), which runs in the statistical package R (v.2.12.1) (http://www.r-project.org), to pick, align, and quantify features (chromatographic events corresponding to specific m/z values and rention times). The software was used with default settings as described (http://metlin.scripps.edu) except for xset (bw = 5) and rector (plottype = "m", family = "s"). The created .tsv file was opened using Excel software and saved as .xls file. The resulting data sheet was normalized to the internal standard and used for further analysis. Metabolite annotation was performed by comparing the accurate mass (m/z) and retention time (Rt) of reference standards in our in-house library and the accurate mass of compounds obtained from the web-based resources such as the Human Metabolome Database (http://www.hmdb.ca/) and The METLIN Metabolite Database (http://metlin.scripps.edu/).

Tissue sample preparation and analysis by lc-tofms

Tissue sample preparation and analysis with LC-TOFMS were performed according to our published report [2, 4, 6]. The breast cancer tissue samples (100 mg) were homogenized on ice in 500 µl mixture of chloroform, methanol and water (1:2.5:1, v/v/v). The samples were then centrifuged at 13,000 rpm for 10 min at 4°C, and a 150-µl aliquot of the supernatant was transferred to an LC sampling vial containing an internal standard (10 µl L-4-chloro-phenylalanine in water, 5 µg/ml). The deposit was re-homogenized with 500 µL of methanol, and a 150-µL aliquot of supernatant was added to the same vial for drying prior to reconstitution with acetonitrile/H2O (6:4, v/v) to a final volume of 500 µl.

The rest of LC-TOFMS operations and statistical procedures were carried out as the LC-TOFMS analysis of plasma/serum samples.

REFERENCES

1. Bao Y, Zhao T, Wang X, Qiu Y, Su M, Jia W: Metabonomic variations in the drug-treated type 2 diabetes mellitus patients and healthy volunteers. J Proteome Res 2009, 8:1623–1630.
2. Qiu Y, Cai G, Su M, Chen T, Zheng X, Xu Y, Ni Y, Zhao A, Xu LX, Cai S et al: Serum metabolite profiling of human colorectal cancer using GC-TOFMS and UPLC-QTOFMS. J Proteome Res 2009, 8:4844–4850.
3. Pan L, Qiu YP, Chen TL, Lin JC, Chi Y, Su MM, Zhao AH, Jia W: An optimized procedure for metabonomic analysis of rat liver tissue using gas chromatography/time-of-flight mass spectrometry. J Pharmaceut Biomed 2010, 52:589–596.
4. Fordahl S, Cooney P, Qiu Y, Xie G, Jia W, Erikson KM: Waterborne manganese exposure alters plasma, brain, and liver metabolites accompanied by changes in stereotypic behaviors. Neurotoxicol Teratol 2012, 34:27–36.
5. Xie G, Zhong W, Zheng X, Li Q, Qiu Y, Li H, Chen H, Zhou Z, Jia W: Chronic Ethanol Consumption Alters Mammalian Gastrointestinal Content Metabolites. Journal of Proteome Research 2013, 12:3297–3306.
6. Chen T, Xie G, Wang X, Fan J, Qiu Y, Zheng X, Qi X, Cao Y, Su M, Xu LX et al: Serum and urine metabolite profiling reveals potential biomarkers of human hepatocellular carcinoma. Mol Cell Proteomics 2011, 10:M110 004945.

Supplementary Table S1: Sources of samples

	No. of samples			
Cancer	City of Hope National Medical Center, California, USA	Ruijin Hospital, Shanghai, China	Ruijin Hospital, Shanghai, China	BioChain Institute,California, USA
Breast (serum)	103		80	
Breast (plasma)	138			
Breast (tissue)				20
Gastric (serum)		114		
Colorectal (serum)		101		
Healthy (serum)	31		70	
Healthy (plasma)	76			
Adjacent normal tissue				20

Supplementary Table S2: List of 225 identified plasma metabolites by GC-TOFMS and LC-TOFMS in breast cancer patients from City of Hope National Medical Center (Training Set)

Compound	Database	Instrument	Mass	Rt (min)
1,2-Dimethylpropanol	NIST	GC-MS	117	4.44
18-Hydroxycorticosterone	HMDB	LC-MS	362.209	19.71
1H-Indole-3-acetate	NIST	GC-MS	202	16.50
1-Hydroxy-1-cyclohexen	NIST	GC-MS	155	5.25
1-Pyrroline-2-carboxylate	HMDB	LC-MS	113.048	3.75
2,2′-Bipyridine	NIST	GC-MS	156	10.65
2,3-Diaminopropionate	Standard	LC-MS	105.065	3.40
2,3-Dihydroxybutanoate	NIST	GC-MS	117	9.56
2,3′-Dipyridyl	NIST	GC-MS	156	11.83
2,4-Dihydroxybutanoate	NIST	GC-MS	103	10.30
2-Amino-6-methylaminohexanoate	NIST	GC-MS	116	14.29
2-Aminobutyrate	Standard	GC-MS	130	6.96
2-Butenedioate	Standard	GC-MS	245	9.34
2-Hydroxycinnamate	HMDB	LC-MS	164.047	11.22
2-Hydroxypyridine	NIST	GC-MS	152	5.01
2-Methyl-butyrate	NIST	GC-MS	159	4.22
2-Oxo-3-methyl-pentanoate	Standard	GC-MS	151	7.07
2-Oxo-4-methylvalerate	Standard	GC-MS	200	7.55
2-Piperidinecarboxylate	NIST	GC-MS	156	9.65
3, 4-Dehydroproline	NIST	GC-MS	208	15.22
3,4-Dihydroxybutanoate	NIST	GC-MS	73	10.55
3,6-Dioxa-2,7-disilaoctane, 2,2,4,7,7-pentamethyl-	NIST	GC-MS	117	4.52
3-Amino-2-Piperidone	NIST	GC-MS	243	10.85
3-Aminosalicylate	Standard	GC-MS	186	11.64
3-Hydroxybutyrate	Standard	GC-MS	147	6.77
3-Hydroxydodecanedioate	HMDB	LC-MS	246.147	18.92
3-Hydroxyoxyisovalerate	NIST	GC-MS	131	7.48
3-Hydroxypyridine	Standard	GC-MS	152	6.41
3-Indolepropionate	HMDB	LC-MS	189.079	20.14
3-Methyl-2-oxo-butanoate	NIST	GC-MS	202	6.43
3-Phosphoglycerate	NIST	GC-MS	211	19.46
3-Pyridylacetate	Standard	LC-MS	138.053	3.86
3-Succinoylpyridine	HMDB	LC-MS	179.058	18.25
4,8-Dimethylnonanoyl carnitine	HMDB	LC-MS	329.257	19.66
4-Aminohippurate	HMDB	LC-MS	194.069	5.57

(*Continued*)

Compound	Database	Instrument	Mass	Rt (min)
4-Deoxypyridoxine	NIST	GC-MS	282	13.67
4-Hydroxy-2-oxoglutarate	HMDB	LC-MS	162.016	3.14
4-Hydroxy-proline	Standard	LC-MS	132.073	3.88
5-Acetylamino-6-formylamino-3-methyluracil	HMDB	LC-MS	226.07	17.22
5-Hydroxyindoleacetate	HMDB	LC-MS	191.058	3.14
5-Hydroxylysine	HMDB	LC-MS	162.1	3.63
5-Hydroxy-tryptophan	NIST	GC-MS	174	21.61
5-Methoxytryptophol	HMDB	LC-MS	191.095	17.93
5-Oxoproline	Standard	GC-MS	156	11.66
5-Phosphoribosylamine	HMDB	LC-MS	229.035	24.78
6-Dehydrotestosterone glucuronide	HMDB	LC-MS	462.225	21.35
6-Deoxy-mannose	NIST	GC-MS	204	17.36
6-Phosphogluconate	Standard	LC-MS	275.012	3.15
9,12-Octadecadienoate (Z,Z)-	Standard	GC-MS	337	19.06
α-Amino isobutyrate	NIST	GC-MS	211	7.21
α-Aminoadipate	NIST	GC-MS	260	12.06
Acetylcarnitine	Standard	LC-MS	204.123	4.14
α-Hydroxyisobutyrate	Standard	GC-MS	131	6.31
Alanine	Standard	GC-MS	116	5.95
Alloisoleucine	Standard	GC-MS	158	8.70
Aminoacetone	HMDB	LC-MS	73.0528	3.82
Aminomalonate	NIST	GC-MS	218	11.05
Androstenedione	HMDB	LC-MS	286.193	18.43
Anthranilate	NIST	GC-MS	208	12.67
Arabinofuranose	NIST	GC-MS	217	14.58
Arachidonate	Standard	GC-MS	91	20.80
Arginine	Standard	LC-MS	175.116	3.49
Asparagine	Standard	GC-MS	116	13.42
Aspartate	Standard	GC-MS	232	11.66
Benzaldehyde	HMDB	LC-MS	106.042	15.04
Benzoate	Standard	GC-MS	179	7.98
Beta-alanine	Standard	GC-MS	174	10.46
beta-D-Glucopyranuronate	HMDB	LC-MS	314.064	3.79
Bilirubin	HMDB	LC-MS	584.263	19.32
Bisnorcholate	HMDB	LC-MS	380.256	22.24
Butyrylcarnitine	HMDB	LC-MS	231.147	16.24
Cadaverine	NIST	GC-MS	174	18.36

(*Continued*)

Compound	Database	Instrument	Mass	Rt (min)
Calcidiol	HMDB	LC-MS	400.334	21.84
Caproate	NIST	GC-MS	173	5.47
Carnitine	Standard	LC-MS	162.107	3.64
Carnosine	Standard	LC-MS	225.106	3.67
Cholesterol	Standard	GC-MS	129	27.22
Choline	Standard	LC-MS	104.105	3.54
cis-2-Methylaconitate	HMDB	LC-MS	188.032	3.08
cis-3-Hexenyllactate	NIST	GC-MS	83	4.67
Citrate	Standard	GC-MS	273	15.10
Citrulline	Standard	LC-MS	176.093	3.66
Creatine	Standard	GC-MS	115	12.11
Cyclohexanone	NIST	GC-MS	58	4.61
Cyclohexyloxy	NIST	GC-MS	157	4.64
Cystathionine	Standard	LC-MS	223.072	3.67
Cysteine	Standard	GC-MS	220	12.08
Cystine	Standard	GC-MS	218	20.30
Decanoate	Standard	GC-MS	229	10.74
Decanoylcarnitine	HMDB	LC-MS	315.241	19.43
Dehydroascorbate	HMDB	LC-MS	174.016	5.48
Delta-hydroxylysine	Standard	LC-MS	163.108	3.63
Deoxycholate	Standard	LC-MS	393.291	23.11
Fructose	Standard	GC-MS	217	15.75
Galactose	Standard	GC-MS	157	15.77
Glucuronate	NIST	GC-MS	333	16.43
Dihydroxyacetone phosphate	HMDB	LC-MS	169.998	3.15
Dodecanoate	Standard	GC-MS	211	13.07
Dodecanoylcarnitine	HMDB	LC-MS	343.272	20.17
Ribofuranose	NIST	GC-MS	217	14.73
Xylose	NIST	GC-MS	217	13.43
Elaidate	Standard	GC-MS	339	19.19
Epinephrine	Standard	LC-MS	184.09	3.60
Epinephrine glucuronide	HMDB	LC-MS	359.122	4.44
Erythrose	NIST	GC-MS	205	14.38
Galactonate	Standard	GC-MS	73	17.12
Glucopyranose	NIST	GC-MS	204	16.82
Glucose 6-phosphate	Standard	LC-MS	259.033	3.61
Glutamate	Standard	GC-MS	246	12.82

(*Continued*)

Compound	Database	Instrument	Mass	Rt (min)
Glutamine	Standard	LC-MS	147.074	3.57
Gluticol	NIST	GC-MS	73	13.58
Glyceraldehyde	Standard	GC-MS	192	7.53
Glyceraldehyde 3-phosphate	Standard	LC-MS	171.004	3.15
Glycerate	Standard	GC-MS	189	9.23
Glycerol	Standard	GC-MS	218	8.45
Glycerolphosphate	Standard	GC-MS	299	14.50
Glycerophosphocholine	HMDB	LC-MS	257.103	3.58
Glycerylphosphorylethanolamine	HMDB	LC-MS	215.056	18.35
Glycine	Standard	GC-MS	174	8.88
Glycol	NIST	GC-MS	147	4.32
Glycolaldehyde	HMDB	LC-MS	60.0211	3.76
Glycylprolylhydroxyproline	HMDB	LC-MS	285.132	22.57
Glyoxylate	NIST	GC-MS	160	4.33
Guanidineacetate	Standard	LC-MS	118.073	3.63
Guanine	HMDB	LC-MS	151.049	5.00
Heneicosanoate	NIST	GC-MS	117	20.52
Hexadecanoate	Standard	GC-MS	117	17.19
Hexanoylcarnitine	HMDB	LC-MS	259.178	18.11
Hexanoylglycine	HMDB	LC-MS	173.105	18.45
Homoanserine	HMDB	LC-MS	254.138	23.27
Homocysteate	HMDB	LC-MS	183.02	17.74
Hordenine	HMDB	LC-MS	165.115	15.36
Hydroxyacetate	Standard	LC-MS	75.0239	4.27
Hydroxycarbamate	NIST	GC-MS	221	9.58
Hypotaurine	Standard	GC-MS	188	13.01
Hypoxanthine	Standard	LC-MS	137.046	5.22
Indoleacrylate	HMDB	LC-MS	187.063	17.31
Inositol	NIST	GC-MS	318	17.38
Isoleucine	Standard	GC-MS	158	8.65
Isopentenyl pyrophosphate	HMDB	LC-MS	246.006	3.11
Isovalerylcarnitine	HMDB	LC-MS	245.163	17.89
Lactate	Standard	GC-MS	117	5.36
Homoserine	NIST	GC-MS	218	13.75
Linoelaidate	HMDB	LC-MS	280.24	23.59
Linoleyl carnitine	HMDB	LC-MS	423.335	21.44
Leucine	Standard	GC-MS	158	8.39

(*Continued*)

Compound	Database	Instrument	Mass	Rt (min)
Methionine	Standard	GC-MS	176	11.62
Ornithine	NIST	GC-MS	142	15.04
Phenylalanyl-L-proline	HMDB	LC-MS	262.132	17.74
Threonate	Standard	GC-MS	292	12.22
Tryptophan	Standard	GC-MS	202	19.39
Tyrosine	Standard	GC-MS	218	16.27
Lysine	Standard	LC-MS	145.09	3.56
Malate	Standard	GC-MS	73	11.26
Maleimide	NIST	GC-MS	154	5.77
Mannitol	Standard	GC-MS	319	16.36
Methionine	Standard	LC-MS	150.058	5.00
Methylcysteine	Standard	GC-MS	218	10.40
Methylguanidine	Standard	LC-MS	74.0583	3.82
Myo-Inositol	Standard	GC-MS	217	18.07
Myristate	Standard	GC-MS	117	15.19
Myristoleate	NIST	GC-MS	256	15.07
N,N-Dimethyl-2-isopropoxyethylamine	NIST	GC-MS	58	4.31
N6-Acetyl-L-lysine	Standard	LC-MS	187.109	4.30
N-Acetyl glucosamine	NIST	GC-MS	274	14.32
N-acetyl-glutamine	Standard	LC-MS	187.082	5.04
N-acetyl-glycine	Standard	GC-MS	144	9.67
N-Acetylneuraminate	NIST	GC-MS	362	21.73
N-formyl-glycine	Standard	GC-MS	160	10.05
Nicotine	NIST	GC-MS	163	5.90
Nicotinate	NIST	GC-MS	180	8.31
Nicotinurate	HMDB	LC-MS	180.053	5.02
Nonanoate	Standard	GC-MS	215	9.49
Norleucine	Standard	LC-MS	132.102	10.48
Octadecanoate	Standard	GC-MS	117	19.40
Octanoate	NIST	GC-MS	201	8.18
Octanoylcarnitine	HMDB	LC-MS	287.21	18.75
oleoylcarnitine	HMDB	LC-MS	425.35	22.00
Olic acid	Standard	GC-MS	339	19.12
Ornithine	Standard	GC-MS	204	15.05
o-Tyrosine	HMDB	LC-MS	181.074	11.22
Oxalate	Standard	GC-MS	73	6.52
Oxaloacetate	Standard	LC-MS	130.992	3.15

(*Continued*)

Compound	Database	Instrument	Mass	Rt (min)
Oxanilate	NIST	GC-MS	147	13.22
Palmitoleate	Standard	GC-MS	129	16.99
Palmitoylcarnitine	Standard	LC-MS	400.342	21.84
Parabanate	NIST	GC-MS	243	10.15
Pentosidine	HMDB	LC-MS	378.202	17.37
Phenylalanine	Standard	GC-MS	218	12.92
Phenylglyoxylate	HMDB	LC-MS	150.032	3.15
Phenyllactate	Standard	LC-MS	165.053	18.33
Phosphoserine	Standard	LC-MS	186.019	3.12
Picolinate	NIST	GC-MS	180	9.03
Progesterone	HMDB	LC-MS	314.225	19.14
Proline	Standard	GC-MS	142	8.74
Proline betaine	HMDB	LC-MS	143.095	4.04
Propionylcarnitine	HMDB	LC-MS	217.131	13.17
Pseudo uridine	NIST	GC-MS	217	20.79
Pyrrole-2-carboxylate	Standard	GC-MS	240	9.52
Pyruvate	Standard	GC-MS	174	5.18
Quinate	HMDB	LC-MS	192.063	4.32
Ribitol	Standard	GC-MS	217	14.11
Sarcosine	NIST	GC-MS	116	6.44
Serine	Standard	GC-MS	204	9.62
Sorbose	NIST	GC-MS	103	14.73
Sphingosine	HMDB	LC-MS	299.282	24.41
Sphingosine 1-phosphate	HMDB	LC-MS	379.249	22.24
Stearoylcarnitine	HMDB	LC-MS	427.366	22.68
Succinate	Standard	GC-MS	247	8.91
Succinyladenosine	HMDB	LC-MS	383.108	3.61
Tetracosahexaenoate	HMDB	LC-MS	356.272	23.51
Tetradecanoylcarnitine	HMDB	LC-MS	371.304	21.00
Thiamine	HMDB	LC-MS	265.112	18.01
Threitol	Standard	GC-MS	217	11.58
Threonine	Standard	GC-MS	219	9.99
Tiglylglycine	HMDB	LC-MS	157.074	15.98
trans-Hexadec-2-enoyl carnitine	HMDB	LC-MS	397.319	21.22
Trimethylamine N-oxide	Standard	LC-MS	76.065	3.67
Undecanoate	HMDB	LC-MS	186.162	21.44
Uracil	Standard	LC-MS	113.022	5.08

(*Continued*)

Compound	Database	Instrument	Mass	Rt (min)
Urea	Standard	GC-MS	189	7.97
Urate	Standard	GC-MS	441	18.10
Valine	Standard	LC-MS	118.092	4.33
Xylitol	NIST	GC-MS	217	13.98
α-Ketoglutarate	Standard	GC-MS	198	12.30

Supplementary Table S3: List of 13 differential metabolites between breast cancer at Stage I+II and breast cancer at Stage III+IV from the plasma samples from Training set and Validation set 1

Metabolite	Database	Instrument	FC	p
Phenylglyoxylate	HMDB	LC-MS	0.94	6.72E-03
Isovalerylcarnitine	HMDB	LC-MS	0.78	1.37E-02
Aspartate	Standard	GC-MS	1.22	1.58E-02
3-Hydroxydodecanedioate	HMDB	LC-MS	0.70	1.60E-02
Methylcysteine	Standard	GC-MS	1.36	1.73E-02
Pseudo uridine	NIST	GC-MS	1.20	1.78E-02
Malate	Standard	GC-MS	1.18	2.38E-02
Threitol	Standard	GC-MS	1.12	3.23E-02
Stearoylcarnitine	HMDB	LC-MS	0.87	3.65E-02
Hypoxanthine	Standard	LC-MS	1.37	4.18E-02
5-Methoxytryptophol	HMDB	LC-MS	0.79	4.19E-02
Octanoate	NIST	GC-MS	1.34	4.56E-02
Ornithine	Standard	GC-MS	1.22	4.84E-02

Supplementary Table S4: List of 83 differential metabolites between breast cancer tissues and adjacent non-tumor tissues from Sample Set 1

Pathway	Compound	Database	Instrument	FC*	*p*
Amino acid metabolism	4-Hydroxyphenylacetate	Standard	GC-MS	0.34	6.2E-09
	N-formyl-glycine	Standard	GC-MS	0.39	1.7E-08
	Pyrrole-2-carboxylate	Standard	GC-MS	0.35	1.8E-08
	Picolinate	Standard	GC-MS	0.37	4.8E-08
	4-Hydroxy-proline	Standard	GC-MS	3.40	6.1E-08
	Glutamate	Standard	GC-MS	2.01	1.1E-06
	Asparagine	Standard	GC-MS	2.18	5.9E-06
	Aspartate	Standard	GC-MS	1.92	7.4E-06
	Tyrosine	Standard	GC-MS	1.69	1.7E-05
	4-Aminobutyrate	Standard	GC-MS	4.73	1.8E-05
	Phenylalanine	Standard	GC-MS	1.69	2.6E-05
	Methionine	Standard	GC-MS	1.73	2.9E-05
	Isoleucine	Standard	GC-MS	1.68	3.3E-05
	5-Oxoproline	Standard	GC-MS	1.58	3.3E-05
	Tryptophan	Standard	GC-MS	1.68	7.0E-05
	Histidine	Standard	GC-MS	1.57	1.0E-04
	Proline	Standard	GC-MS	1.52	1.2E-04
	Methylcysteine	Standard	GC-MS	1.48	1.5E-04
	Aminomalonate	NIST	GC-MS	2.42	3.8E-04
	Carnosine	Standard	LC-MS	0.42	3.9E-04
	Serine	Standard	GC-MS	1.46	4.0E-04
	Threonine	Standard	GC-MS	1.45	4.3E-04
	Cysteine	Standard	GC-MS	2.02	9.9E-04
	Leucine	Standard	GC-MS	1.39	1.0E-03
	2-Aminobutyrate	Standard	GC-MS	1.91	1.4E-03
	3-Methylhistidine	HMDB	LC-MS	0.36	2.0E-03
Carbohydrate metabolism	Citrate	Standard	GC-MS	0.28	1.6E-05
	Myoinositol	Standard	GC-MS	0.47	4.0E-05
	Xylulose	Standard	GC-MS	1.54	4.3E-05
	Sucrose	Standard	GC-MS	0.32	7.5E-05
	Xylose	Standard	GC-MS	1.96	3.6E-04
	N-acetyl-D-glucosamine	Standard	GC-MS	2.00	4.4E-04
	Rhamnose	Standard	GC-MS	1.74	5.1E-03

(*Continued*)

Pathway	Compound	Database	Instrument	FC*	*p*
Lipid metabolism	Nonadecanoate (C19:0)	NIST	GC-MS	0.35	3.3E-09
	Glycerol	Standard	GC-MS	0.40	5.1E-09
	Caprate (C10:0)	Standard	GC-MS	0.45	2.4E-08
	Hexanoate (C6:0)	Standard	GC-MS	0.45	4.7E-07
	Adrenate (22:4(n-6))	NIST	GC-MS	0.58	4.9E-06
	Nonanoate	Standard	GC-MS	0.45	7.6E-06
	Phosphoethanolamine	Standard	GC-MS	3.21	1.7E-05
	Palmitoleate (C16:1)	Standard	GC-MS	0.29	1.9E-05
	Myristate (C14:0)	Standard	GC-MS	0.31	4.6E-05
	Squalene	Standard	GC-MS	0.14	5.0E-05
	Laurate(C12:0)	Standard	GC-MS	0.30	6.7E-05
	LysoPC (16:0)	HMDB	LC-MS	0.35	1.1E-04
	Cholesterol	Standard	GC-MS	0.48	1.2E-04
	Linolate (18:2(n-6))	Standard	GC-MS	0.24	1.2E-04
	Hexadecanoate (C16:0)	Standard	GC-MS	0.41	1.7E-04
	Octanoate	NIST	GC-MS	0.60	2.2E-04
	Palmitoylcarnitine	Standard	LC-MS	4.21	9.5E-04
	Octadecanoate (C18:0)	Standard	GC-MS	0.53	9.6E-04
	Glyceraldehyde 3-phosphate	Standard	GC-MS	2.50	1.4E-03
	Hexanoylcarnitine	Standard	LC-MS	3.85	1.7E-03
	1-Stearoylglycerol	Standard	GC-MS	0.65	1.8E-03
	3-Dehydrosphinganine	HMDB	LC-MS	3.72	1.9E-03
	Arachidyl carnitine	HMDB	LC-MS	12.09	3.8E-03
	Palmitin	Standard	GC-MS	0.60	3.8E-03
Nucleic acid metabolism	Cytosine	NIST	GC-MS	0.45	5.2E-08
	Ribose	NIST	GC-MS	2.54	3.5E-07
	Uracil	Standard	GC-MS	2.95	2.9E-06
	Guanosine	Standard	GC-MS	3.81	3.3E-05
	Orotidine	Standard	GC-MS	4.42	4.8E-05
	Dihydrouracil	Standard	GC-MS	3.52	8.4E-05
	Hypoxanthine	Standard	GC-MS	1.54	2.5E-04
	8-Hydroxy-deoxyguanosine	HMDB	LC-MS	3.75	4.5E-04
	Ribonate	NIST	GC-MS	1.62	9.1E-04
	Uridine	Standard	GC-MS	2.10	2.5E-03
Other	3-Hydroxypyridine	Standard	GC-MS	0.37	7.9E-09

(*Continued*)

Pathway	Compound	Database	Instrument	FC*	*p*
	2-Hydroxypyridine	Standard	GC-MS	0.35	1.2E-08
	Phenol	Standard	GC-MS	0.28	1.4E-08
	Cyclohexanol	NIST	GC-MS	0.30	1.6E-08
	3-Octenoate	NIST	GC-MS	0.34	3.2E-08
	Phosphate	NIST	GC-MS	0.51	2.5E-07
	Benzoate	Standard	GC-MS	0.37	3.1E-06
	Threonate	Standard	GC-MS	0.36	3.0E-05
	Diphosphorate	NIST	GC-MS	0.36	1.2E-04
	Methylsuccinate	Standard	GC-MS	0.54	1.3E-04
	Hydroxyacetate	Standard	GC-MS	0.43	1.3E-04
	1,4-Butanediamine	Standard	GC-MS	3.28	4.2E-04
	alpha-Tocopherol (vitamin E)	Standard	GC-MS	0.37	1.5E-03
	Pantothenate (vitamin B5)	Standard	GC-MS	1.84	1.8E-03
	Spermidine	Standard	LC-MS	2.65	2.0E-03
	5-Hydroxyindoleacetate	HMDB	LC-MS	3.16	2.4E-03

* Fold change (FC) with *a* value more than 1 indicates a relatively higher concentration in the breast tumor samples, while *a* value less than 1 means a relatively lower concentration compared to the controls.

Supplementary Table S5: Multiple reaction monitoring transitions and MS parameters for the LC–MS/MS analysis

Analyte	Parent (m/z)	Daughter (m/z)	Auto Dwell	Dwell (s)	Cone (v)	Collision (v)
Aspartate	134.07	73.99	0	0.5	20	14
^{13}C-labeled aspartate	138.07	75.99	0	0.5	22	14

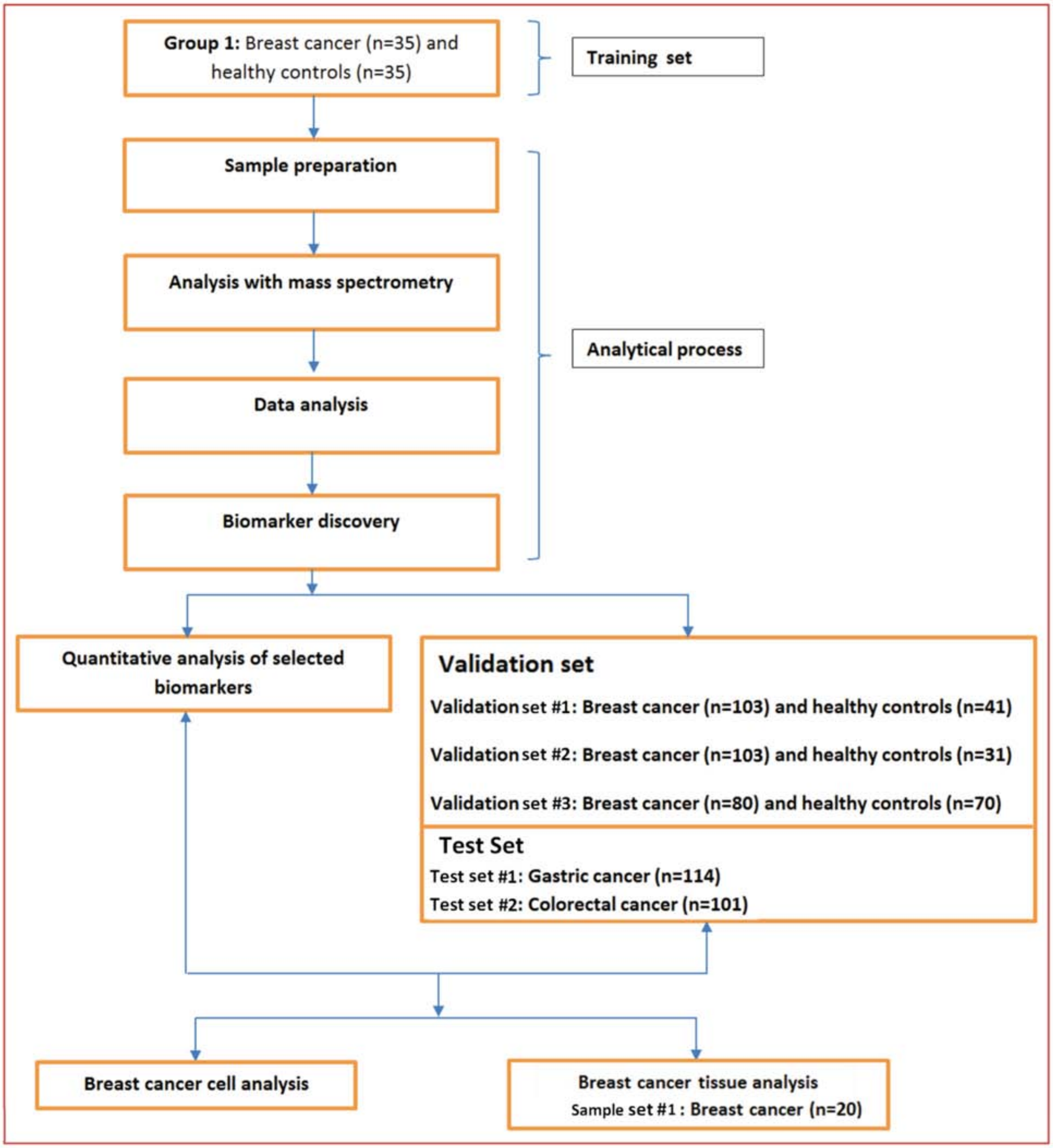

Supplementary Figure S1: Flow chart of experimental design.

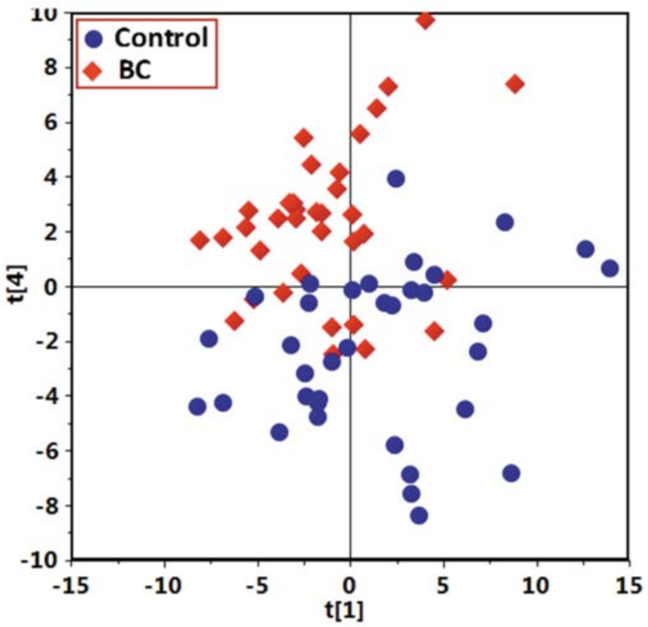

Supplementary Figure S2: The scores plot of the PCA model of the Training Set samples. The PCA model was constructed using the plasma data from 35 breast cancer (BC) patients (red diamonds) and 35 healthy controls (blue dots).

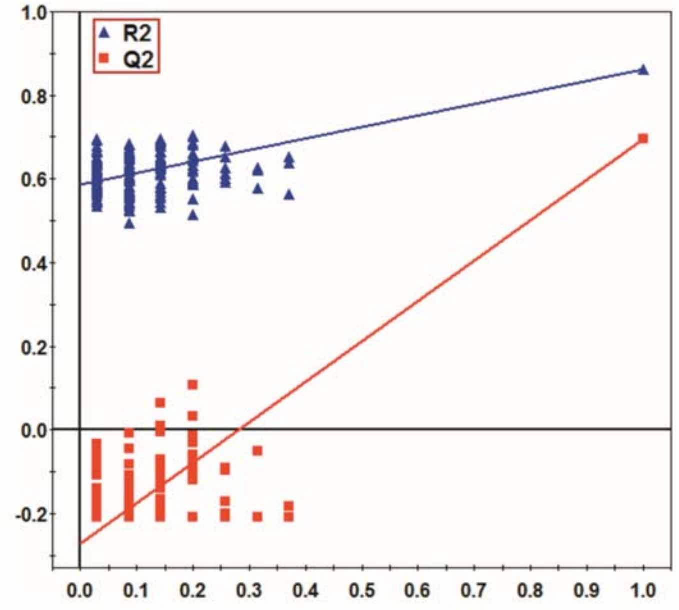

Supplementary Figure S3: The permutation testing (200 times) result.

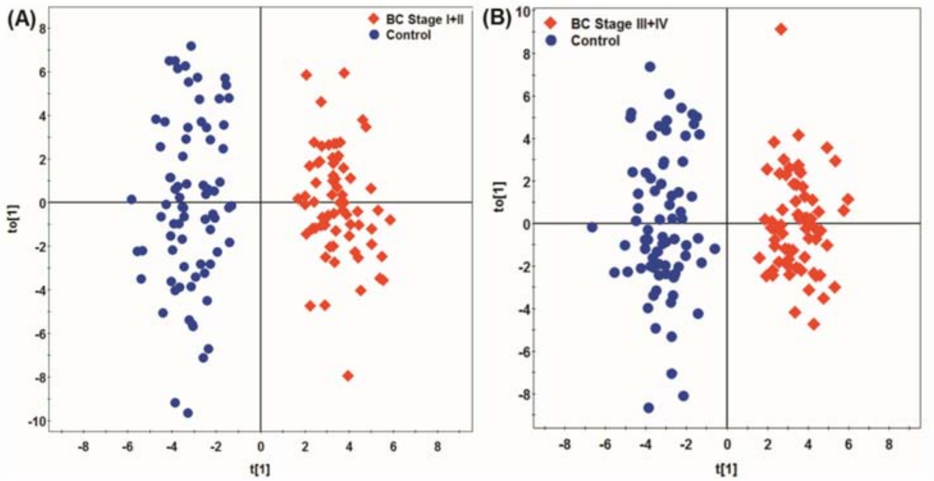

Supplementary Figure S4: The scores plot of the OPLS-DA model based on the 225 identified plasma metabolites between 69 breast cancer patients at Stages I and II and 76 healthy controls. A. and 69 breast cancer patients at Stages III and IV and 76 healthy controls **B.** from City of Hope (Training set and Validation set 1).

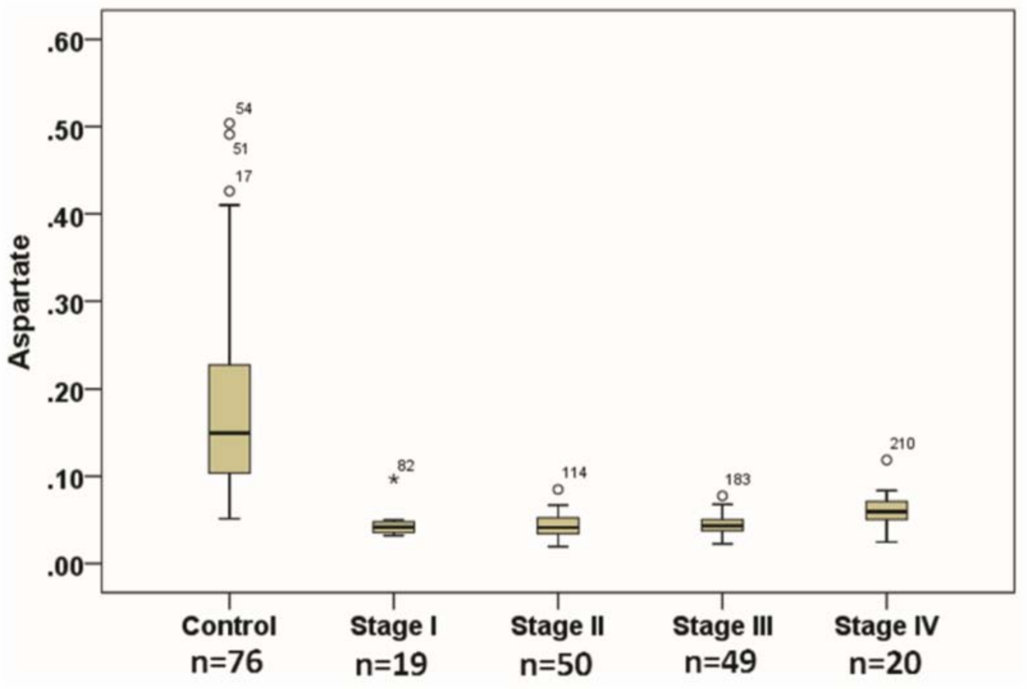

Supplementary Figure S5: Box plot of the plasma aspartate levels in healthy controls (*n* = 76) and breast cancer patients at Stages I (*n* = 19), II (*n* = 50), III (*n* = 49), and IV (*n* = 20).

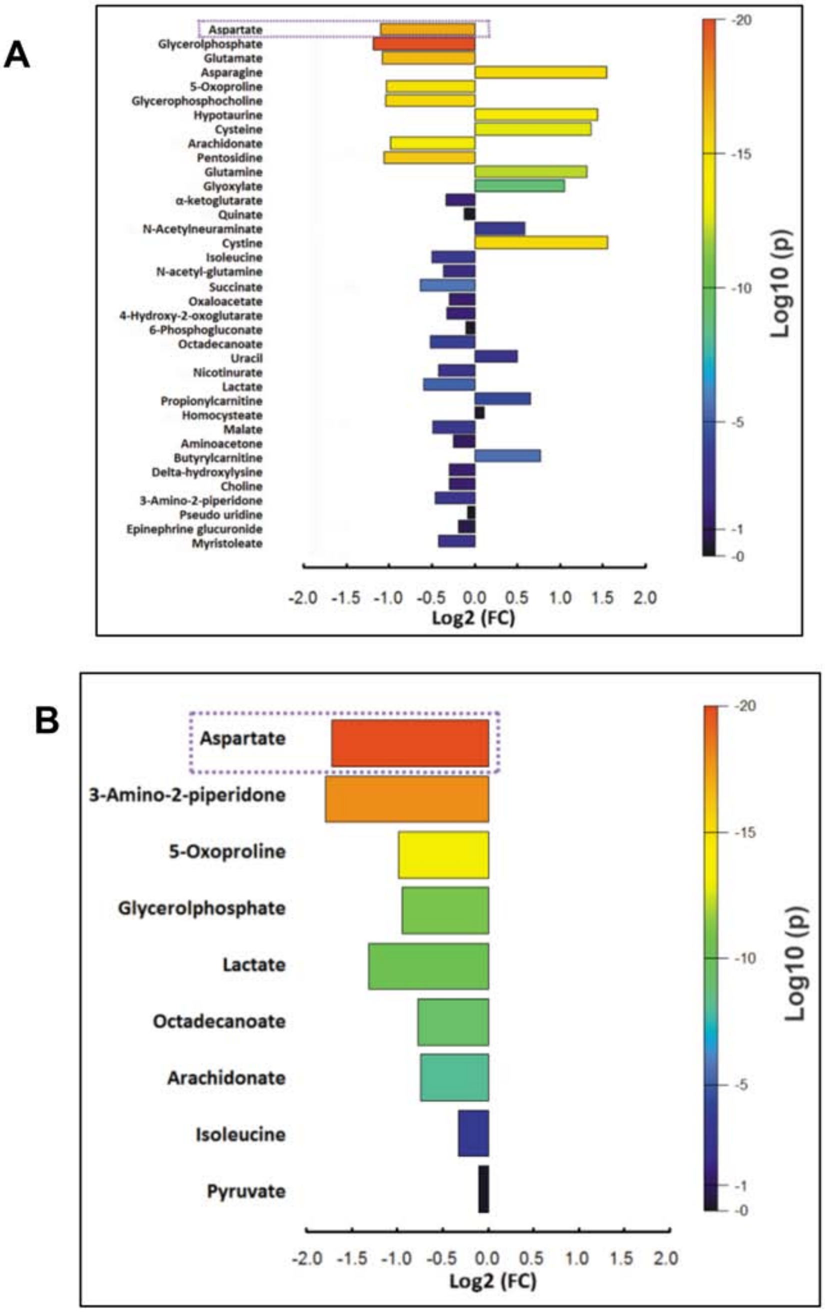

Supplementary Figure S6: Bar plot of the metabolic differences in serum between breast cancer patients and healthy controls from A. Validation set 2 and B. Validation Set 3. A fold change value was calculated for each metabolite by taking the ratio of the mean intensities in breast cancer and healthy controls. Each bar representing a fold change value was colored to indicate its corresponding *p*-value and thereby specify the statistical significance in all subjects (see color scale).

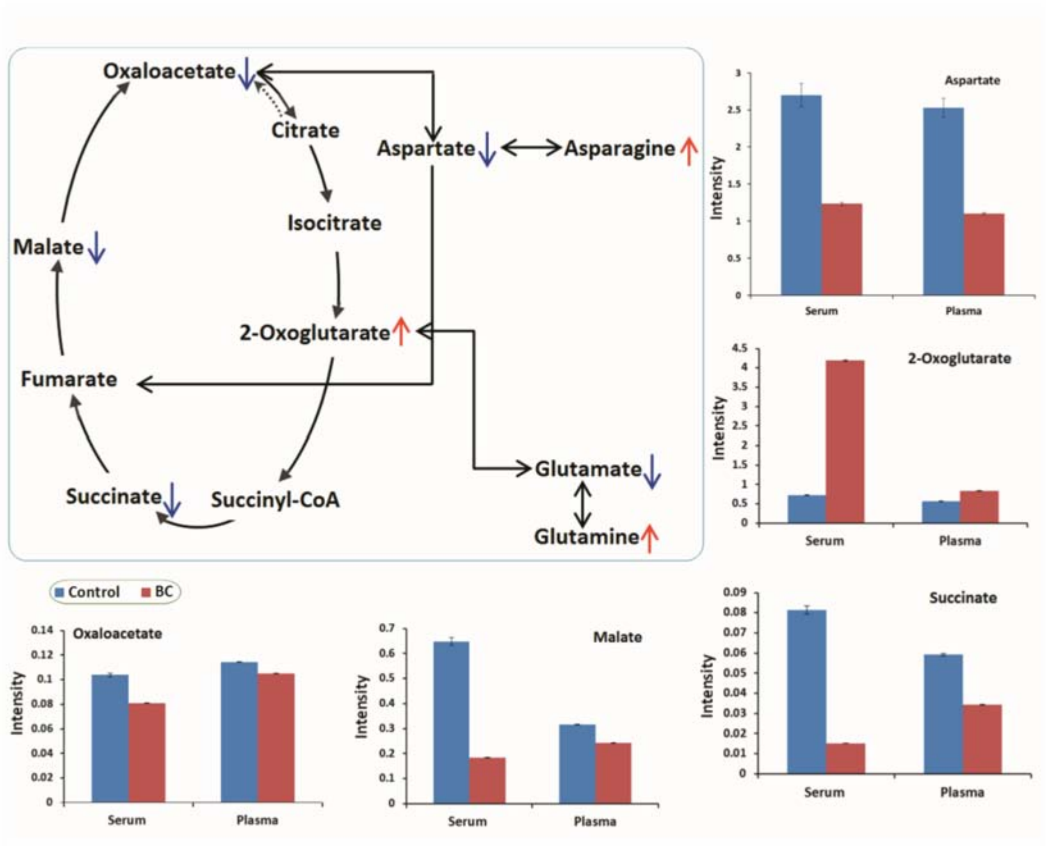

Supplementary Figure S7: Altered metabolic pathways in breast cancer (BC) patients compared to those in the healthy controls and bar plot of five representative differential metabolites between breast cancer patients and healthy controls. The bar plots show the fold change (y axis) of each metabolite in the pathway as the ratio of the concentration in the breast cancer patient to that in the control in serum and plasma (x axis).

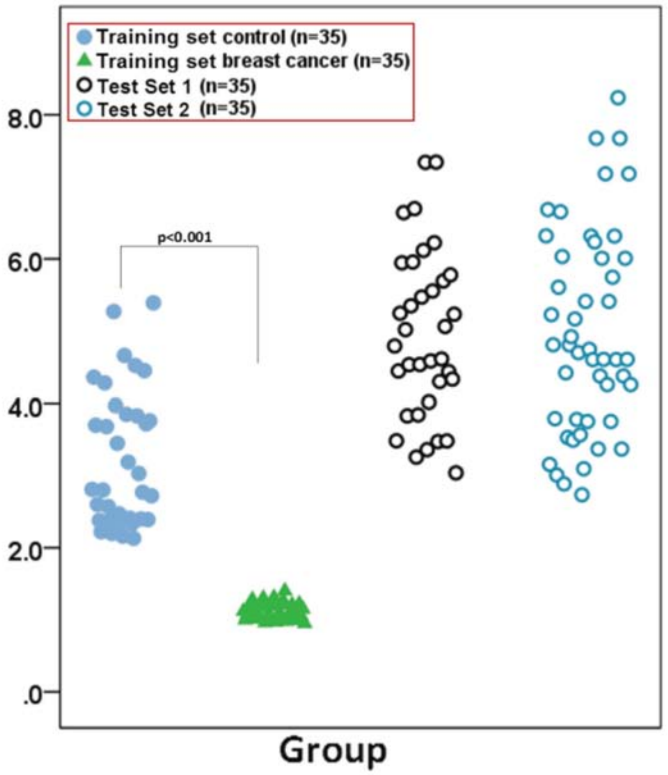

Supplementary Figure S8: Distributions of aspartate concentration in different samples (n = 35 in each group). *P*-value over a group denotes statistical significance of differences between each group member and healthy controls.
